# Geospatial Analysis of Neighborhood Environmental Stress in Relation to Biological Markers of Cardiovascular Health and Health Behaviors in Women: Protocol for a Pilot Study

**DOI:** 10.2196/29191

**Published:** 2021-07-22

**Authors:** Kosuke Tamura, Kaveri Curlin, Sam J Neally, Nithya P Vijayakumar, Valerie M Mitchell, Billy S Collins, Cristhian Gutierrez-Huerta, James F Troendle, Yvonne Baumer, Foster Osei Baah, Briana S Turner, Veronica Gray, Brian A Tirado, Erika Ortiz-Chaparro, David Berrigan, Nehal N Mehta, Viola Vaccarino, Shannon N Zenk, Tiffany M Powell-Wiley

**Affiliations:** 1 Social Determinants of Obesity and Cardiovascular Risk Laboratory, Cardiovascular Branch National Heart, Lung, and Blood Institute National Institutes of Health Bethesda, MD United States; 2 Office of Biostatistics Research, Division of Intramural Research National Heart, Lung, and Blood Institute National Institutes of Health Bethesda, MD United States; 3 Health Behaviors Research Branch, Behavioral Research Program Division of Cancer Control and Population Sciences National Cancer Institute, National Institutes of Health Shady Grove, MD United States; 4 Section of Inflammation and Cardiometabolic Diseases, Cardiovascular Branch, Division of Intramural Research National Heart, Lung, and Blood Institute National Institutes of Health Bethesda, MD United States; 5 Department of Epidemiology Rollins School of Public Health Emory University Atlanta, GA United States; 6 Department of Medicine School of Medicine Emory University Atlanta, GA United States; 7 National Institute of Nursing Research National Institutes of Health Bethesda, MD United States; 8 Intramural Research Program National Institute on Minority Health and Health Disparities National Institutes of Health Bethesda, MD United States

**Keywords:** wearables, global positioning system, ecological momentary assessment, accelerometer, biomarkers of stress, mobile phone

## Abstract

**Background:**

Innovative analyses of cardiovascular (CV) risk markers and health behaviors linked to neighborhood stressors are essential to further elucidate the mechanisms by which adverse neighborhood social conditions lead to poor CV outcomes. We propose to objectively measure physical activity (PA), sedentary behavior, and neighborhood stress using accelerometers, GPS, and real-time perceived ecological momentary assessment via smartphone apps and to link these to biological measures in a sample of White and African American women in Washington, DC, neighborhoods.

**Objective:**

The primary aim of this study is to test the hypothesis that living in adverse neighborhood social conditions is associated with higher stress-related neural activity among 60 healthy women living in high or low socioeconomic status neighborhoods in Washington, DC. Sub-aim 1 of this study is to test the hypothesis that the association is moderated by objectively measured PA using an accelerometer. A secondary objective is to test the hypothesis that residing in adverse neighborhood social environment conditions is related to differences in vascular function. Sub-aim 2 of this study is to test the hypothesis that the association is moderated by objectively measured PA. The third aim of this study is to test the hypothesis that adverse neighborhood social environment conditions are related to differences in immune system activation.

**Methods:**

The proposed study will be cross-sectional, with a sample of at least 60 women (30 healthy White women and 30 healthy Black women) from Wards 3 and 5 in Washington, DC. A sample of the women (n=30) will be recruited from high-income areas in Ward 3 from census tracts within a 15% of Ward 3’s range for median household income. The other participants (n=30) will be recruited from low-income areas in Wards 5 from census tracts within a 15% of Ward 5’s range for median household income. Finally, participants from Wards 3 and 5 will be matched based on age, race, and BMI. Participants will wear a GPS unit and accelerometer and report their stress and mood in real time using a smartphone. We will then examine the associations between GPS-derived neighborhood variables, stress-related neural activity measures, and adverse biological markers.

**Results:**

The National Institutes of Health Institutional Review Board has approved this study. Recruitment will begin in the summer of 2021.

**Conclusions:**

Findings from this research could inform the development of multilevel behavioral interventions and policies to better manage environmental factors that promote immune system activation or psychosocial stress while concurrently working to increase PA, thereby influencing CV health.

**International Registered Report Identifier (IRRID):**

PRR1-10.2196/29191

## Introduction

### Background

Promoting physical activity (PA) is a critical public health goal because insufficient PA participation affects all age groups and various racial and ethnic groups in the United States (US) [1]. Approximately 10% of deaths are attributed to insufficient PA in the US [2], and engaging in regular PA (ie, meeting the PA guidelines of at least 150 min/week of at least moderate intensity PA) can reduce the risk of numerous chronic diseases (eg, cardiovascular disease [CVD]) [3]. Furthermore, in 2018, the newly released Physical Activity Guidelines for Americans, 2nd edition documented that regular PA has immediate health benefits, including lowering blood pressure, increasing sleep quality, decreasing anxiety, and improving cognitive function and insulin sensitivity [4]. Despite the well-demonstrated PA benefits, most US adults do not engage in sufficient PA, when measured by accelerometers [5,6]. In particular, African American adults have a lower PA level than their White counterparts, with 7.7% of African American adults and 8.2% of White adults meeting the PA guidelines [6].

The application of a multilevel social-ecological framework to determine changes in the environment and policy that would promote PA participation has been supported by authoritative US health institutions and international organizations over the past two decades [7-9]. This conceptual framework identifies relevant factors at multiple levels, ranging from genetic, intrapersonal, interpersonal, social, and cultural, to environmental factors [10]. The key principle of the social-ecological model posits that each level can impact behavior, and individuals can impact and are influenced by their environment, particularly when considering psychosocial effects [11]. Recent interventions are increasingly focusing on the role that the neighborhood social environment (eg, poverty, social disorder, and crime [12]) may play as a source of stressors that shape low levels of PA at the population level [8,13].

Although the neighborhood social environment is an important factor in promoting PA [13], a key limitation in neighborhood social environment research is that the majority of such research systematically focuses on residential areas (ie, home) when investigating relationships between objectively measured environmental exposures via geographic information system (GIS) and PA [13]. However, individuals are generally mobile and engage in daily activities that are not restricted to places close to residential areas (eg, workplace) [14]. This generates a geospatial mismatch between exposures to the neighborhood social environment and locations where health behaviors occur [13,15,16]. Previous studies have also used self-reported PA, resulting in potential recall and social desirability bias [17].

When coupled with the neighborhood social environment as a source of stressors, psychosocial factors, such as chronic stress and depression, have an inverse relationship with PA and ultimately CVD [18]. Furthermore, a recent study in adults without CVD and our research in African American women in Washington, DC, demonstrated that amygdala activity (ie, chronic stress–related neural activity) assessed via 18-fluorodeoxyglucose (FDG) positron emission tomography-computed tomography (PET/CT) were significantly associated with subsequent CVD events [19] and vascular inflammation, a subclinical marker of atherosclerosis [20,21]. In addition, another recent study showed that neighborhood-level socioeconomic status (SES) was inversely associated with amygdala activity and arterial inflammation [22]. These findings are important for validating the relationship between chronic stress because of neighborhood factors and CVD events. However, the major limitations of the previous study were that they did not account for individual-level SES in the analytic models. Instead, they used neighborhood-level SES as a proxy for individual-level SES and examined the associations between neighborhood SES and amygdala activity. Furthermore, participants were chosen from a clinical database, which was not representative of the general US adults, and they were predominantly White adults [22]. Further research is needed to elucidate the associations between psychosocial factors, amygdala activity, and biological markers of adverse cardiac events in diverse populations and to assess both individual- and neighborhood-level SES.

### Objectives

To address these limitations, researchers have increasingly used an objective PA monitor (ie, accelerometer) linked to data from GPS units to track locations where PA occurs [23,24]. GPS units are often used to quantify an individual’s daily activity space (ie, defined as locations where individuals travel or move throughout the day) [23-28]. Ecological momentary assessment (EMA) also helps researchers to better understand psychosocial factors (eg, stressors and mood) in real time and psychosocial-environment contexts for health behaviors [29], such as PA and sedentary behavior. Therefore, the simultaneous use of three distinct methods (accelerometer, GPS, and EMA) reduces recall bias and the geospatial mismatch between exposures to the neighborhood social environment and PA [13]. In our study, we plan to measure both individual- and neighborhood-level SES among a diverse sample of adult women.

This pilot research will address current gaps in understanding the determinants of health disparities for populations from both high- and low-SES neighborhoods in Washington, DC, by applying geospatial tools and methods (ie, accelerometer, GPS, and EMA) and by linking to biomarkers of stressors. This research is innovative because it will apply geospatial tools and methods for tracking individual daily mobility and examine their linkage with biological measures, integration of neighborhood social measures for more comprehensive and objective assessment of factors that may lead to stress-related neural activity and poor CV outcomes. Therefore, the aims of this pilot study are as follows:

Aim 1: To test the hypothesis that living in adverse neighborhood social environment conditions is associated with higher stress-related neural activity among 60 healthy women living in high- or low-SES areas in Washington, DC.

Sub-aim 1: To test the hypothesis that the association is moderated by objectively measured PA via an accelerometer and psychosocial factors via EMA.

Aim 2: To test the hypothesis that residing in adverse neighborhood social environment conditions is related to differences in vascular function.

Sub-aim 2: To test the hypothesis that the association is moderated by objectively measured PA and psychosocial factors via EMA.

Aim 3: To test the hypothesis that adverse neighborhood social environment conditions are related to differences in immune system activation.

### Implications

This geospatial pilot study has a strong impact because the associations between neighborhood social contexts and stress-related neural activity (ie, marker of chronic stress–related neural activity) are highly understudied. This study is also novel because the linkages between neighborhood factors, detailed immune markers related to amygdala activity, and vascular function are limited. In addition, this geospatial pilot study will be among the first to include innovative geospatial technologies and tools to further characterize exposure to adverse neighborhood contexts within an individual’s daily mobility and link these data to an individual’s stress and mood through EMA data. Findings from this research could accelerate the development of multilevel behavioral interventions and environmental policies to better manage environmental factors that promote psychosocial stress or interventions that increase PA, which in turn can promote cardiovascular health.

## Methods

### Study Design

**Figure 1:**
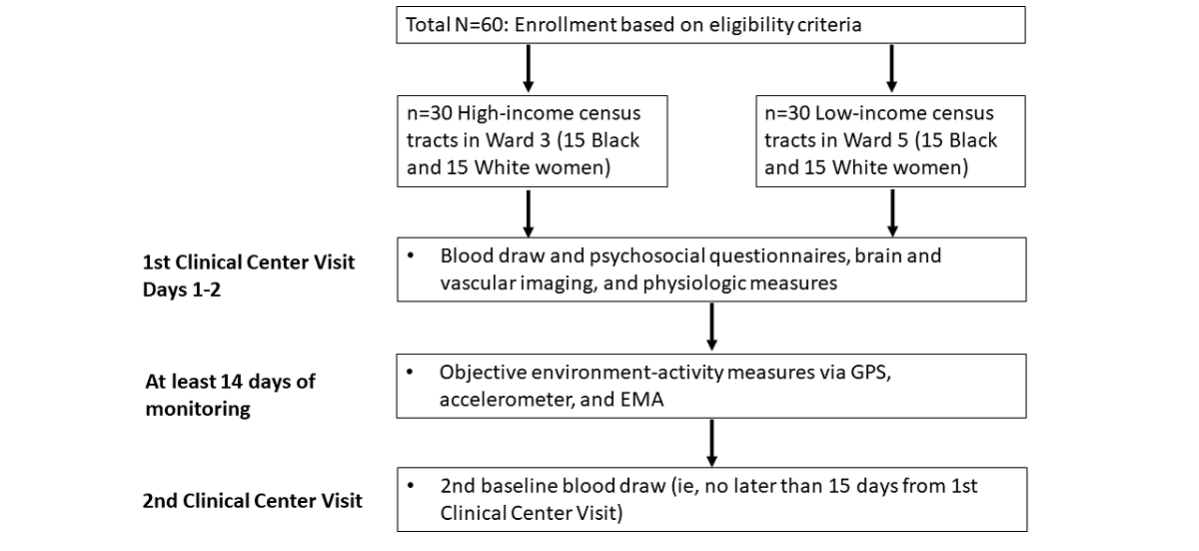
Flowchart of events.

This is a cross-sectional study designed to investigate the impact of neighborhood environment on cardiovascular health and PA in African American and White women residing in Washington, DC, neighborhoods. Study participants will first visit the National Institutes of Health (NIH) Clinical Center where baseline health information will be collected and three devices will be distributed (accelerometer, GPS, and EMA app; Figure 1). After 14 days, participants will return to the Clinical Center for a final blood draw and cardiovascular examination. The NIH Institutional Review Board (IRB) approved this study, and this study has been registered on ClinicalTrial.gov (NCT04014348).

### Study Participants

A sample of women (at least n=30; 15 White women and 15 African American women) will be recruited from higher SES census tracts within a 15% range of the median household income in Ward 3 (median household income=US $126,184) [30]. The other participants (at least n=30; 15 White women and 15 African American women) will be recruited from lower SES census tracts within a 15% range of the median household income in Ward 5 (median household income=US $68,375) [31]. Participants from Wards 3 and 5 will be matched based on age, race, and BMI.

### Eligibility

Individuals eligible for this protocol will meet the following inclusion criteria: (1) a healthy White female or healthy Black female of African descent; (2) must be aged between 19 and 45 years; (3) must not have any chronic health condition, including lung disease or active infection; (4) must be living in Washington, DC, Wards 3 or 5; (5) must have access to a smartphone; (6) must be able to provide informed consent; and (7) must speak English. Individuals who meet the following criteria will be excluded from this study: (1) pregnant or breastfeeding; (2) physically unable to perform PA for any reason; (3) weight changes greater than 20% over the past 3 months; (4) obesity (measured BMI≥30.0 kg/m^2^); (5) high or low blood pressure; (6) diabetes; (7) history of mental illnesses, treated with medication and therapy; (8) history or evidence of hyper or hypothyroidism; (9) current medication use for chronic illness; (10) HIV; and (11) food allergies or highly restrictive diets that may prevent the ability to consume a controlled metabolic diet.

### Recruitment

Recruitment strategies include (1) distribution of recruitment flyers targeting potential participants in public locations throughout Wards 3 and 5, concentrating on areas in or near the targeted census tracts; (2) inclusion of study information on ClinicalTrials.gov, NIH Search the Studies, and a dedicated recruitment website on the Clinical Center Office of Patient Recruitment website (Current Protocols area); (3) use of ResearchMatch. for identification of healthy volunteers meeting criteria; (4) use of NIH (National Heart, Lung, and Blood Institute [NHLBI]/Clinical Center) social media accounts—Facebook, Twitter, and Craigslist with IRB-approved messages; and (5) distribution of recruitment messages on NIH listservs. This study was approved by the NIH Intramural IRB (NCT 04014348). Before enrollment in this study, our trained research staff will obtain written informed consent from all participants.

### Devices

Participants will receive an accelerometer (ie, objective activity monitor; ActiGraph wGT3X-BT) to measure PA, sedentary time, and sleep duration for at least 14 days. The ActiGraph accelerometer has been previously used to objectively assess PA [23,24], sedentary time [23,24], and sleep duration [32] among adults. The data will be collected at 1-minute epochs. Two distinct cut-points based on approaches by Troiano [5] and Matthews [33] will be used to determine the intensity of PA. Participants will be instructed to wear the monitor on their dominant wrist with a wristband at all times, except when bathing or swimming. A valid day of accelerometer monitoring is defined as ≥10 hours of wear time [33]. The time-stamped recordings from the accelerometers will be linked with GPS data.

Participants will also receive a GPS unit (ie, tracking device, Qstarz BT-Q1000eX GPS Logger). The GPS unit receives a satellite signal to identify latitude and longitude coordinates for a given period. GPS units will monitor participants’ daily activities via locations where they travel throughout the day for at least 14 monitoring days. GPS units will also record distance, speed, elevation, and time. The data will be collected at 1-minute intervals. This device has been used successfully in a recent study [34]. This GPS device is small and can be worn on a belt or placed in a backpack or bag. It will provide a timestamp and GPS coordinates. A valid day of GPS tracking is defined as ≥10 hours of wear time [35].

We will use a smartphone app to record EMA data via a mobile phone; these methods have been previously used for adults [36,37]. We will use two approaches for participants to record their PA, neighborhood environment, and stress. The first is an event contingency assessment. With this assessment, participants will be expected to record within the smartphone app when they engage in a certain behavior (eg, PA) within 15 minutes of the event occurring. The other type of assessment involves random survey prompts. Participants will respond to a series of questions on each day of the week. They will receive EMA random prompts for each assessment for that day (up to three random prompts for each assessment [morning, afternoon, and evening] and a maximum of nine prompts each day). The survey may take approximately 2-3 minutes to complete each time. Each item assesses the type of neighborhood context for the physical environment (eg, home, work, and outdoor), vegetation (amount of trees), traffic (amount of traffic), and safety (feeling safe and violence). Mood (positive and negative affect) and stressors (eg, daily hassles) from the EMA survey will be used specifically as psychosocial factors for each sub-aim. Previously validated EMA surveys and protocols for adults will be used in the proposed study [36,37].

### Compliance

EMA compliance rates will be based on answered surveys divided by the total number of scheduled EMA surveys for each participant. We will also monitor participation rate, missing data, latency period (defined as the time between receiving EMA prompts and the items answered), and attrition rate [38]. In additionally, we will follow up each participant daily, irrespective of the compliance rate, to ensure that they do not have issues with device use. Basic statistical analyses will be performed to better understand participant compliance, such as compliance rate, missingness, participation rate, and latency period. In addition, as an exploratory analysis, the missingness of EMA surveys will be examined using pattern mixture random-effects modeling [39]. Furthermore, mindless EMA responses to surveys may occur. To address this issue, we have selected survey items that are crucial to our study aims to minimize participants’ burden.

### Preliminary Study

Before this pilot study, we conducted the DC CV Health and Needs Assessment (DC-CHNA) study (NCT019227783) to investigate bio-psychosocial and neighborhood conditions resulting in poor CV health. The objective of the previous work was to determine ways to use mobile health technology to promote CV health for populations in lower SES DC Wards 5, 7, and 8 [40-44]. First, the DC-CHNA created a community advisory board, the DC Cardiovascular Health and Obesity Collaborative (DC-CHOC), to give feedback on developing and implementing the DC-CHNA and subsequent community-based projects. The DC-CHOC consists of representatives from the DC faith-based community, US government agencies, academia, and health advocacy organizations, all of whom are devoted to addressing obesity and CV health in at-risk DC communities [44-47]. In this study, 11 participants from DC-CHNA were asked to carry GPS units and use the EMA app and then test the feasibility of both a GPS device and an EMA app for African American women.

### Schedule of Events

Participants will visit the NIH Clinical Center where we will instruct them on how to use the devices and measurement tools (accelerometer, GPS, and EMA; Table 1). During the inpatient visit, we will conduct a cardiovascular examination, draw blood, and conduct a vascular stiffness test. Participants will also undergo 18F-FDG-PET/CT testing to measure chronic stress–related neural activity (ie, amygdala brain activity) and vascular inflammation. On the first day of the inpatient visit, study volunteers will follow a controlled eucaloric diet containing 55% carbohydrate, 15% protein, and 30% fat and providing 150% of their estimated resting energy needs in preparation for testing to accurately measure energy expenditure on inpatient day 2. Resting energy expenditure has not been included in the aims of this study. However, this measure can be evaluated as a moderator for the sub-aims of aims 1 and 2.

**Table 1:** Schedule of events.

Events				First Clinical Center visit					At least 14-day monitoring			2nd Clinical Center visit
				Day 1		Day 2						
Recruitment				Ongoing		Ongoing		Ongoing			Ongoing	
Informed consent				✓								
Vital signs				✓								
Anthropometric measures				✓								
Clinical blood testing				✓							✓	
Cardiovascular examination				✓							✓	
Blood pressure				✓							✓	
Blood glucose				✓							✓	
Hemoglobin A_1c_				✓							✓	
Lipid panel				✓							✓	
Resting energy expenditure						✓						
18-fluorodeoxyglucose positron emission tomography-computed tomography						✓						
Pulse wave velocity				✓								
**Survey assessments^a^**												
	Sociodemographic characteristics			✓							✓	
	Medical history			✓							✓	
	**Health behaviors**			✓							✓	
		Self-rated health	✓							✓		
		Smoking status	✓							✓		
		Alcohol use	✓							✓		
		General physical activity level	✓							✓		
		Illicit drug use	✓							✓		
		Sleep duration and quality	✓							✓		
		Dietary intake	✓							✓		
	Perceived Stress Scale			✓							✓	
	Center for Epidemiological Studies-Depression Scale			✓							✓	
	Hamilton Anxiety Rating Scale			✓							✓	
	Adverse Childhood Experiences			✓							✓	
	Life Orientation Test-Revised (a measure of optimism)			✓							✓	
	MacArthur Scale of Subjective Social Status			✓							✓	
	Perceived Ethnic Discrimination Questionnaire Community Version			✓							✓	
	Positive and Negative Affect Schedule			✓							✓	
	Superwoman Schema			✓								
	Perceived Neighborhood Environment			✓							✓	
	COVID-19 surveys			✓								
**Physical activity, sedentary behavior, sleep, and neighborhood disorder assessments**												
	Physical activity and sedentary behavior via accelerometers							✓				
	Sleep duration via accelerometers							✓				
	GPS monitoring							✓				
	Ecological momentary assessment							✓				
	Engagement with the DC Cardiovascular Health and Obesity Collaborative Community Advisory Board			Ongoing		Ongoing		Ongoing			Ongoing	

^a^The surveys can be completed at the first or second clinical visit and will not be repeated.

During their baseline visit, participants will also undergo anthropometric measurements, including height, weight, waist circumference, and hip circumference for weight-related outcomes; complete questionnaires to measure demographics and medical history, health behaviors (eg, PA [48]), perceived neighborhood measures, psychosocial factors (eg, perceived general stress [49]), and COVID-19 questionnaires [50,51]. The majority of the participants will complete the survey questionnaires during the baseline visit (visit 1). For some reason, if participants do not complete the surveys during visit 1, we allow them to complete surveys during the 2nd visit to avoid protocol deviation. A second blood draw will be performed within 14 days from the first draw, as a second baseline to account for the variability of sensitive biological measures. This study follows a tiered compensation format where total compensation depends on the number of assessments the participant completes.

After the baseline assessment (ie, clinical visit days 1 and 2), participants will wear an accelerometer to objectively measure PA and carry a GPS unit to assess locations where they engage in PA for at least a 14-day period [52]. Concurrently, participants will carry a smartphone to receive EMA surveys that have been previously validated to measure psychosocial factors, including mood and stress, for a 14-day period. Subsequently, we will create GIS-derived neighborhood environmental variables, psychosocial variables (EMA), PA or sedentary behavior, and diet outcomes using data from the surveys, EMA, accelerometers, and GIS unit. We will then examine associations between environmental exposures and biological measures, psychosocial factors, PA or sedentary behavior, and dietary intake.

### Measures

#### Outcomes

Participants will visit the NIH Clinical Center for 18-FDG PET/CT to measure stress-related neural activity. In brief, we will assess amygdala uptake as published [19,53]. Immune activation testing from collected blood will be performed within the NIH Clinical Center.

Two measures of vascular inflammation and function will be used (Table 2). First, the target-to-background ratio for aortic vascular inflammation will be measured through the whole body FDG PET/CT to examine amygdala activity [54]. Second, the pulse wave velocity and augmentation index as a measure of large-vessel vascular function will be determined by the Sphygmocor system (AtCor Medical), a noninvasive tool [55]. The noninvasive measure of vascular function has been validated and used widely.

**Table 2:** Biological measures, physical activity, residential and GPS activity space, and ecological momentary assessment measures.

Measures			Description
**Outcomes**			
	Stress-related neural activity	Amygdala activity (18F-FDG^a^ PET/CT^b^)	
	Vascular inflammation and function testing	Vascular inflammation: arterial Inflammation (18F-FDG PET/CT) Vascular function: pulse wave velocity and augmentation index (Sphygmocor)	
	Immune system activation	Flow cytometry-based characterization of immune cell populations and their receptor expression profile (two flow cytometry panels) [56]: (1) CD3, CD14, CD15, CD16, CD19, CD42b, CD45, CD56, CD203c, and CD193 and (2) CD3, CD14, CD16, CD56, CD98 heavy chain, CD64, CCR2^c^, CCR5, and TLR2^d^	
	Immune cell function	Natural killer cell function profiling by detecting degranulation and cytolytic activity [57,58] Monocyte function profiling [59], including ability of monocytes to perform chemotaxis and migration, as well as determination of inflammasome activity	
	Biomarker-based immune system activation	Cytokine and chemokine profiling [60] eg, TNF-α^e^, IL-6f, IL-10, IL-8, IL-1β, IFN-α^g^, IFN-γ, MCP-1^h^, VEGF-A^i^, IL-RA^j^, IL-18, TGF-β^k^ Stress-induced neurotransmitter profiling, including epinephrine, norepinephrine, dopamine, and cortisol [61]	
**Exposures**			
	Residential and GPS activity space (model: BT-Q1000XT)	Neighborhood Deprivation Index [62,63] based on US census [62], social disorder based on virtual neighborhood audits [41,64], crime rate around GPS activity space based on police crime report [65], and modified retail food environment index. All measures will be created based on GPS activity space [66] Count and density of parks, gyms, and recreation facilities [67] around GPS activity space	
**Moderators**			
	PA^l^ via accelerometer or survey	A minute-by-minute PA (A^m^), daily mean moderate-to-vigorous PA minutes (A), total PA minutes (A), active transportation PA, and leisure-time PA minutes [46] (S^n^)	
	Psychosocial factors via ecological momentary assessment	Perceived neighborhood social environment [68,69]; mood states [70], perceived stress [49], and daily hassles [71]	

^a^FDG: fluorodeoxyglucose.

^b^PET/CT: positron emission tomography-computed tomography.

^c^CCR2: C-C chemokine receptor type 2.

^d^TLR2: toll like receptor 2.

^e^TNF-α: tumor necrosis factor α

^f^IL-6: interleukin-6.

^g^IFN-α: interferon-α.

^h^MCP-1: monocyte chemoattractant protein-1.

^i^VEGF-A: vascular endothelia growth factor-A.

^j^IL-RA: receptor antagonist.

^k^TGF-β: transforming growth factor β.

^l^PA: physical activity.

^m^A: assessed by accelerometer.

^n^S: assessed by survey.

The outcomes for immune cell activation studies will be the proportion of each detected immune cell, their receptor expression, and platelet adhesion (eg, proportion of classical clusters of differentiation, such as CD14+CD16- monocytes of all monocytes, or proportion of natural killer cells of all CD45+ cells; Table 2). Immune cell function for purified and isolated monocytes and natural killer cells will also be determined for each participant. For instance, the ability of monocytes to perform chemotaxis, migrate toward the monocyte chemoattractant protein-1 gradient, will be determined based on a chemotaxis coefficient, as well as the activity of the inflammasome by measurement of interleukin (IL)-1β and IL-18 release by enzyme-linked immunosorbent assay upon appropriate stimulation. In biomarker profiling, plasma or serum levels of the proposed cytokines, chemokines, and stress-related neurotransmitters, and hormones will be measured as concentrations in pg/mL.

#### Exposures

**Figure 2:**
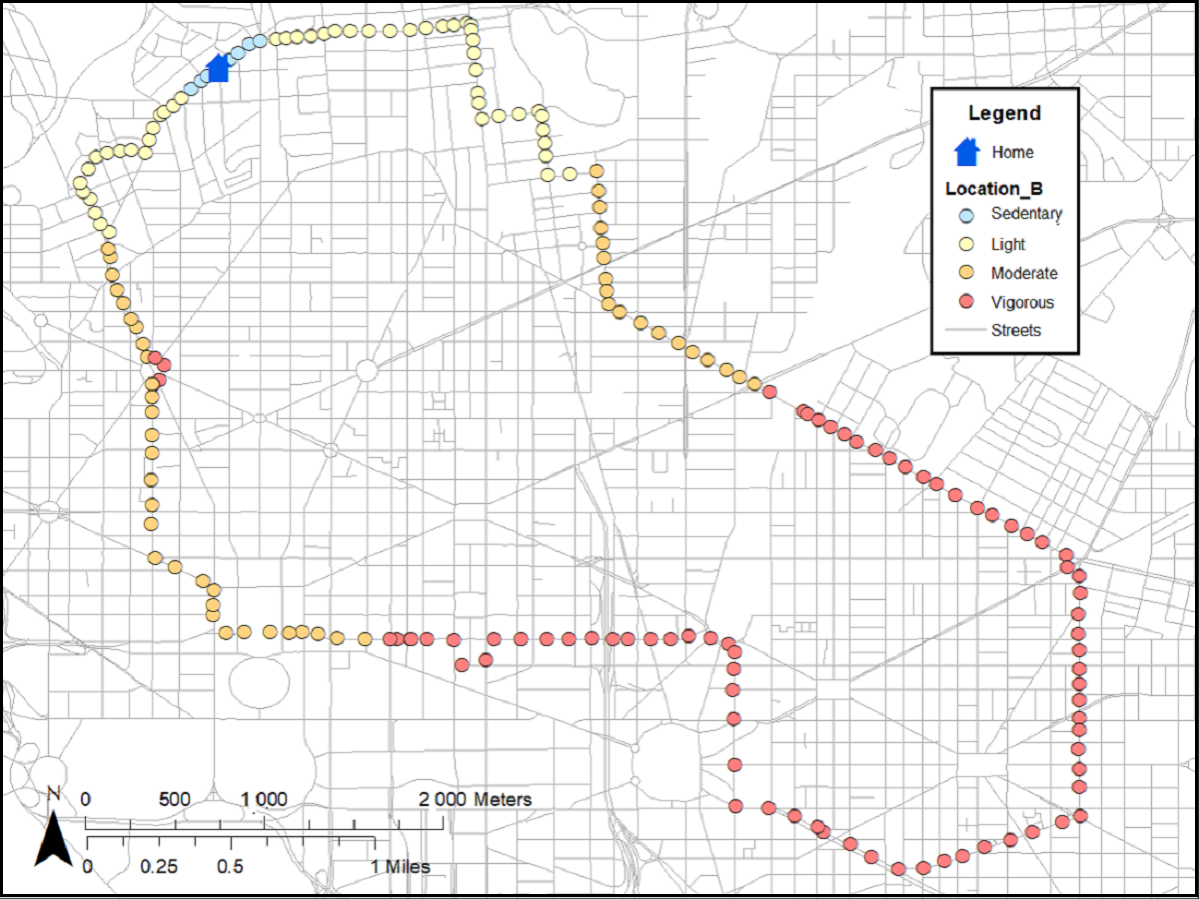
Minute-by-minute accelerometer data linked to GPS coordinates. Note: each point represents a minute of physical activity, ranging from sedentary to vigorous intensity levels.

**Figure 3:**
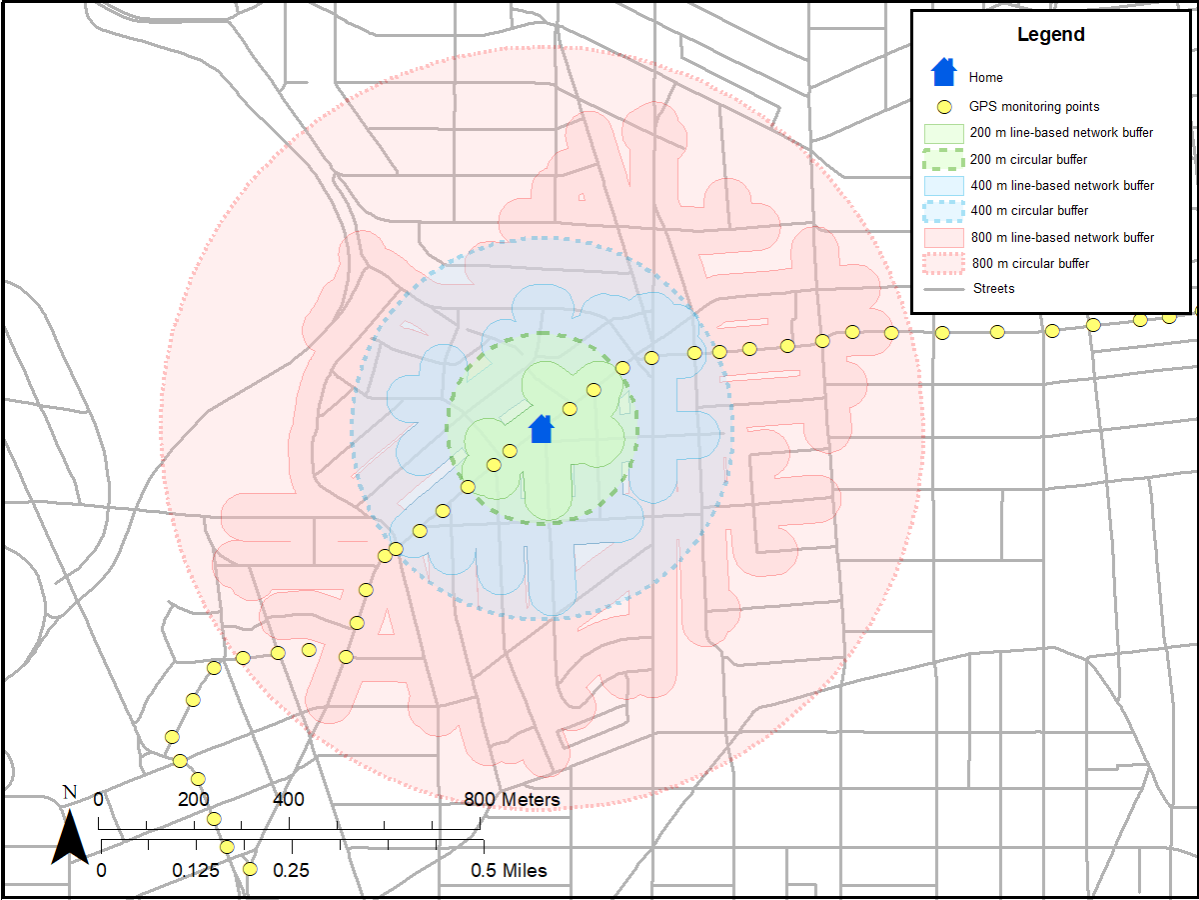
Residential buffers around participants’ home based on circular and line-based road network buffers.

**Figure 4:**
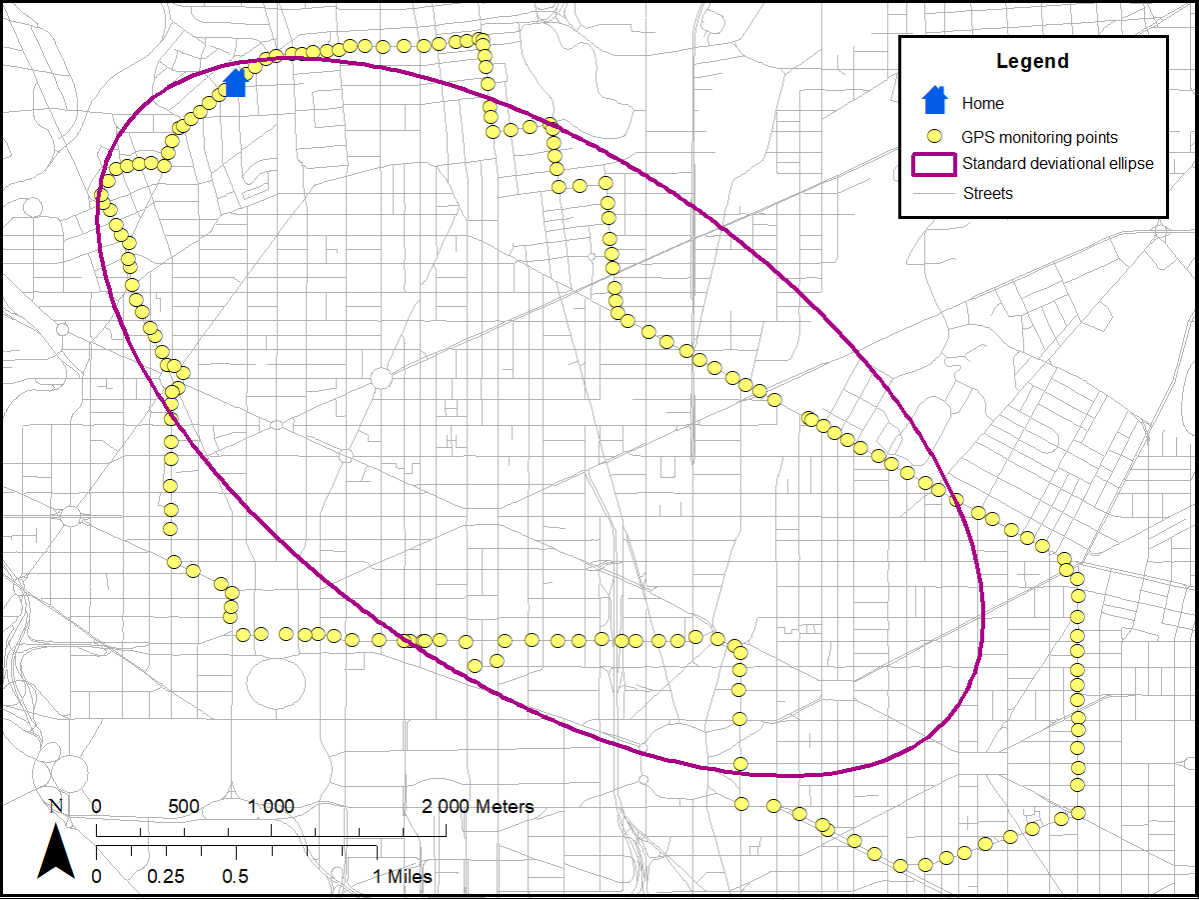
GPS activity space buffers based on SD ellipses.

**Figure 5:**
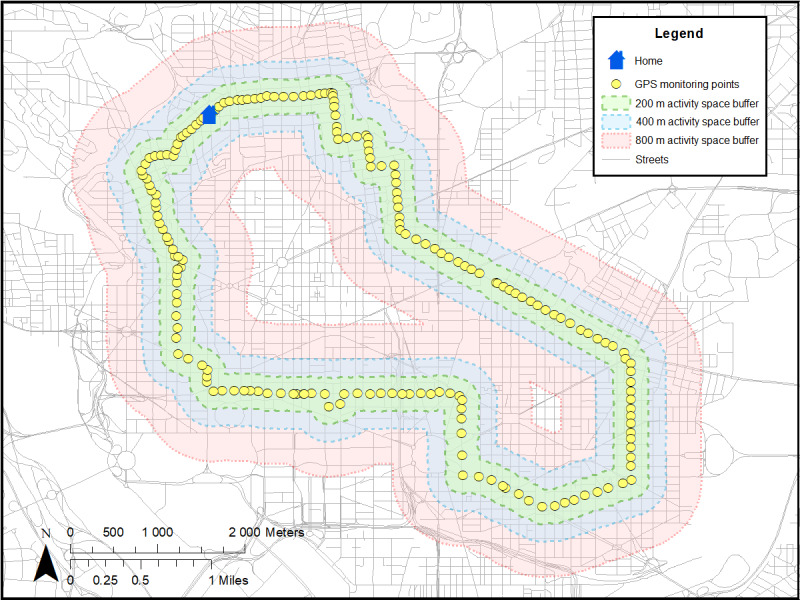
GPS activity space buffers based on individuals’ daily paths.

Two distinct types of neighborhood exposures will be created (Table 2): (1) residential neighborhood variables around participants’ homes [25-27] and (2) GPS activity space (ie, places where participants travel throughout the day) [25-27]. Each type of measure will be linked to accelerometer data with respective time and measured intensity of PA levels (Figure 2; Table 2). Both residential and GPS activity space measures will include neighborhood poverty (eg, neighborhood deprivation index from US census [62,63]), physical disorder (by virtual neighborhood audits [41,64]), police-reported crime (from DC databases [65,72]), and the modified retail food environment index (defined as the ratio of healthy to unhealthy food stores) [73]. In addition, parks, gyms, and recreation facilities will be used to create the count and density of these PA facilities to better understand how participants engage in PA [67]. As a measure of feasibility and practicality, we used 11 participants from a protocol (ClinicalTrials.gov identifier NCT03288207) to create residential buffers around participants’ homes (ie, circular [25-27] and line-based road network buffers; Figure 3 [74]) and GPS activity space buffers based on SD ellipses [66,75] (Figure 4) and individuals’ daily paths (Figure 5) [66]. Figures 2-5 were based on hypothetical data to show each different buffer and buffer type (actual study participant data are not shown). Some points occurring around residential areas will be weighted.

**Figure 6:**
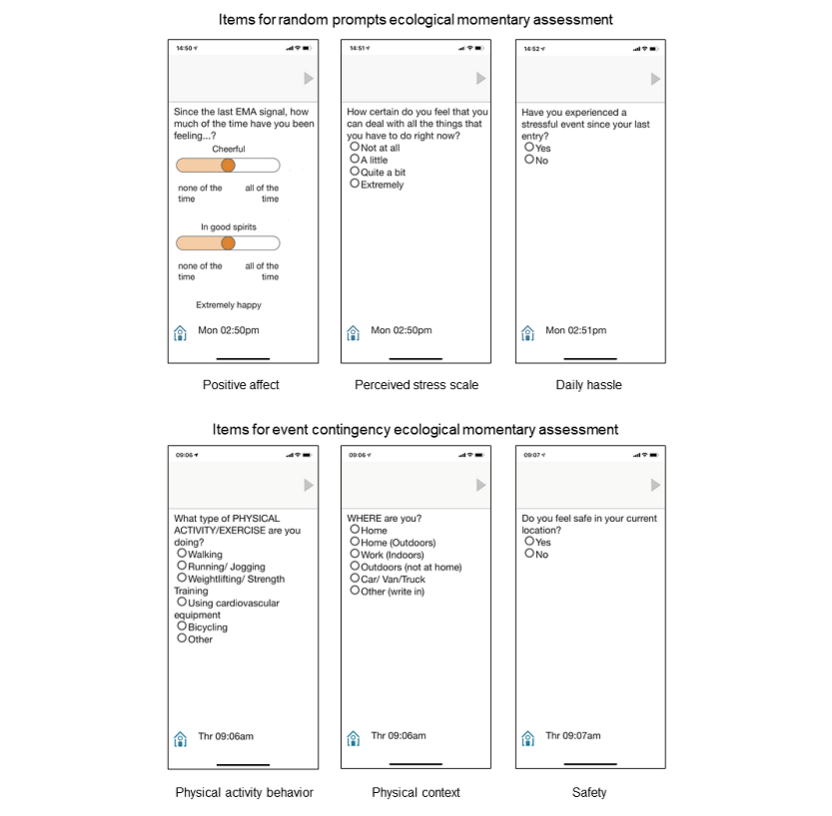
Selected random prompts ecological momentary assessment and event contingency ecological momentary assessment. EMA: ecological momentary assessment.

In Table 3, real-time EMA measures will include perceived neighborhood social environment (eg, violence and safety), mood states (positive or negative affect [70]), perceived stress (eg, stressed and irritated [49]), and daily hassles (eg, work load [71]) with three random prompts within defined timeframes (7-9 AM, 11 AM-1 PM, and 5-7 PM) throughout the day (Table 3; Figure 6) [36]. Participants will initiate their own EMA recording when they start engaging in a certain activity (ie, event contingency; Table 3; Figure 6) [68,69]. The event contingency approach is based on a self-initiated response to behaviors and contexts. Participants will be instructed on how to initiate the EMA recording and respond to a certain activity and when to initiate their EMA recording. For example, when participants commute to work by car, they initiate it by themselves and record their mode of activity in the smartphone app. EMA measures will be merged with GPS activity space measures with the respective timestamps. Before the full pilot study, we tested GPS units and EMA using a smartphone app (ilumivu, Inc) to identify any potential barriers to use.

**Table 3:** Random prompts and event contingency items for ecological momentary assessment.

Variable			Item		Response options
**Items for random prompts**					
	Positive and negative affect [70]	“Since the last EMA^a^ signal, how much of the time did you feel...?” Cheerful In good spirits Extremely happy Calm and peaceful Satisfied Full of life So sad Nervous Restless or fidgety Hopeless Worthless Everything was an effort		Very slightly or not at all A little Moderately Quite a bit Extremely	
	Perceived stress scale [49]	“How certain do you feel that you can deal with all the things that you have to do right now?”		Not at all A little Quite a bit Extremely	
	Perceived stress scale [49]	“How confident do you feel about your ability to handle all of the demands on you right now?”		Not at all A little Quite a bit Extremely	
	Daily hassles [71]	“Have you experienced a stressful event since your last entry?”		Yes or no	
	Daily hassles [71]	“Have you experienced a stressful or problematic social interaction since last entry?”		Yes or no	
**Items for event contingency** **[68,69]**					
	Physical activity behavior	“What type of physical activity/exercise are you doing?”		Walking Running or jogging Weightlifting or strength training Using cardiovascular equipment Bicycling Other (write in)	
	Physical context	“WHERE are you?”		Home (indoors) Home (outdoor) Work (indoor) Outdoors (not at home) Car, van, or truck Other (write in)	
	Physical context	“WHERE are you AT HOME?” (Indoors)		Bedroom Family or living room Kitchen Garage Other (write in)	
	Physical context	“WHERE are you AT HOME?” (Outdoors)		Pool Deck, patio, or balcony Yard Driveway Other (write in)	
	Physical context	“Where are you OUTDOORS?” (Outdoors not at home)		Park or trail Road Sidewalk Parking lot Other (write in)	
	Vegetation	“How many TREES AND PLANTS are there in the area where you are right now?”		No trees or plants A few trees and plants Some trees and plants A lot of trees and plants	
	Traffic	“How much TRAFFIC is on the closest street to where you are right now?”		No traffic A little traffic Some traffic A lot of traffic	
	Litter	“How much LITTER or GARBAGE is on the ground where you are right now?”		No litter A little litter Some litter A lot of litter	
	Safety	“Do you feel safe in your current location?”		Yes or no	
	Safety	“Is violence a problem in your current location?”		Yes or no	

^a^EMA: ecological momentary assessment.

### Merging Multiple Data

Complete master data will be created based on multiple data sources, including PA via accelerometers (ie, 1-minute epochs), GPS units (1-minute epochs), and EMA via a smartphone app (3 times/day). First, each recording of the GPS points will be linked to the respective time-stamped accelerometer data for each minute. Then, EMA data (three times through morning, afternoon, and evening assessments) will be linked to the corresponding GPS and accelerometer data. The complete data will then be collapsed by hours, time of day (morning, afternoon, and evening), and days.

### Statistical Analysis

To test aim 1 hypothesis, we will first test the differences in stress-related neural activity by Wards 3 and 5. Subsequently, we will examine associations between each neighborhood social environment variable based on residential and GPS-derived measures as well as EMA measures separately and stress-related neural activity, adjusting for all covariates. Covariates will include sociodemographic variables (eg, age, race [White or Black adults], and individual-level income) and health-related variables (eg, PA). Each model will be assessed based on the value of the Akaike information criterion (AIC) to select the most parsimonious model. Third (sub-aim 1), after determining a statistically significant neighborhood exposure variable, we will test for an interaction between the specific neighborhood exposure variable and objectively measured PA and psychosocial factors (a potential moderator). If the interaction term is significant, we will stratify the association by high or low objectively measured PA based on the mean and by high or low psychosocial factors (eg, perceived stress scale scores) based on the mean.

To test aim 2 hypothesis, we will first test the differences in the measures of vascular inflammation and function (Table 2) by Wards 3 and 5. Second, we will examine associations between each neighborhood social environment variable based on residential and GPS-derived measures as well as EMA separately and each vascular measure, adjusting for all covariates. The same covariates will be used as in aim 1. Each model will be assessed based on the value of the AIC to select the best-fit model. Third (sub-aim 2), after we identify a statistically significant exposure variable, we will test for an interaction between the specific exposure variable and objectively measured PA and psychosocial factors. If the interaction term is significant, we will then stratify the association by high or low objectively measured PA based on the mean and by high or low psychosocial factors (eg, perceived stress scale scores) based on the mean.

To test aim 3 hypothesis, we will first test the differences in immune cell activation, immune cell function, and biomarker-based immune activation (Table 2) by Wards 3 and 5. Second, we will examine associations between each neighborhood social environment variable based on residential and GPS-derived measures as well as EMA separately and the measures of immune system activation, adjusting for all covariates. The covariates are described in aim 1. Each generalized linear mixed model will be assessed based on the value of the AIC to select the best-fit model.

### Sample Size Calculation

This pilot study (n=30 in each of the two groups) will examine the differences in biological measures to perform power calculations for a larger study. This will also serve as a feasibility study to determine the feasibility of applying GPS, GIS, and EMA for a larger sample of adults. A sample size of 30 per group allows for estimation of the SD of the amygdala FDG in each group, with an upper bound on the SD 20% larger than the estimate, based on the normal distribution large sample 90% upper confidence bound of:

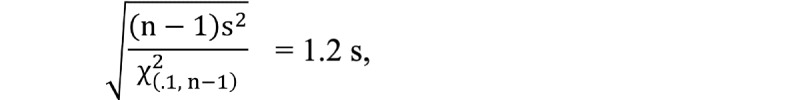



where 
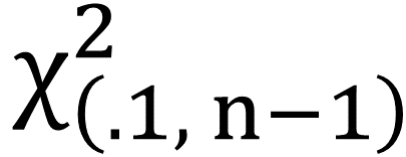
 is the 10% quantile of the chi-squared distribution with n−1 degrees of freedom and s is the SD estimate. We plan to recruit approximately 90 participants, assuming that approximately 20% of participants will be lost to follow-up during the study.

### Plans for Data Management

The NHLBI IRB has approved this protocol. The data with patient identifiers will be securely stored in the eHealth and data management systems at the NIH, which are protected by passwords and data encryption. These data will be shared only with approved members of the research team. The entire data set for the participants included the clinical information, GPS or accelerometer data, data through the EMA mobile app, and survey data.

A Food and Drug Administration–approved biospecimen tracking inventory system will be used to securely store all collected biological specimens at the NIH. The NIH IRB will review and approve any future testing of undefined biospecimens in the protocol before any data collection. The participants are allowed to refuse future use.

This research is scheduled to begin in 2021, with plans for completion by 2023. However, because of the COVID-19 pandemic, it may be delayed depending on the current pandemic situation. Participants who receive unintended consequences from study participation will be treated according to best practices under the NIH Clinical Center. This study is low risk; thus, it does not require a data monitoring committee. The principal investigator will monitor accrual and safety data. The protocol will be reviewed and monitored annually by the IRB and the NHLBI Office of the Clinical Director’s Protocol Audit Team, along with any amendments requiring IRB approval. The results and conclusions of this research will be disseminated to community members through DC-CHOC meetings and a quarterly newsletter as well as at national and international conferences and in peer-reviewed publications.

## Results

### Feasibility of GPS Use

We tested the feasibility of using a GPS device and an EMA app with 11 participants from a PA-related study among African American women (ClinicalTrials.gov identifier NCT03288207). The mean age of the women was 56 years, and most of the participants had at least a high school education (Table 4). The majority of participants had a total household income of US $60,000-89,999; were employed part-time; and resided in Prince George’s County, Maryland.

**Table 4:** Sociodemographic characteristics of participants (N=11).

Participant characteristics			Values
Age (years), mean (SD)			56.3 (12.3)
Gender (female), n (%)			11 (100)
**Race, n (%)**			
	Black or African American	11 (100)	
**Education, n (%)**			
	College degree	4 (36)	
	Graduate or professional school degree	3 (27)	
	Some college degree	2 (18)	
	High school diploma or Tests of General Education Development	1 (9)	
	Some graduate or professional school	1 (9)	
**Total household income (US $), n (%)**			
	20,000-59,000	3 (27)	
	60,000-89,999	5 (46)	
	90,000-99,999	0 (0)	
	≥100,000	3 (27)	
**Employment status, n (%)**			
	Yes, full-time	3 (9)	
	Yes, part-time	4 (12)	
	No, retired	3 (9)	
	Other	1 (3)	
**Participant’s residence (county, Maryland or Ward, Washington, DC), n (%)**			
	Prince George’s County	6 (54)	
	Ward 5	3 (27)	
	Ward 7	2 (18)	

Mean areas varied by the size of the buffers (200 m, 400 m, and 800 m) and types of buffers based on residential (circular and line-based road network buffers) and GPS activity space (Table 5). As expected, the areas for a line-based road network buffer were smaller than that for a circular buffer. Areas for residential buffers (circular and network buffers) were also smaller than the GPS activity space areas. We plan to explore different sizes and types of buffers.

**Table 5:** Mean areas for participants’ residential (circular and network) and GPS activity space buffers (N=11).

Type of buffer	Size (km^2^), mean (SD)		
	200 m	400 m	800 m
Circular buffer	0.13 (0)	0.50 (0)	2.01 (0)
Network buffer^a^	0.05 (0.02)	0.17 (0.08)	0.64 (0.33)
GPS activity space	20.48 (8.09)	53.32 (20.77)	123.02 (46.72)

^a^On the basis of a line-based road network buffer around the residence of a participant.

### Protocol Approval

Full support for this study has been received from the NIH IRB (ClinicalTrial.gov identifier NCT04014348).

## Discussion

### Principal Findings

This pilot study would contribute significantly to scholarship regarding the association between neighborhood social environment (eg, poverty and crime) and stress-related neural activity, a measure of chronic stress–related neural activity. This is important because there is limited research on this topic. This study is also novel because the relationships between neighborhood social environment conditions, detailed immune markers linked to physiological stress–related neural activity response, and vascular function are extremely understudied.

### Lessons Learned From Feasibility of GPS Use

When 11 participants from our study (ClinicalTrials.gov identifier NCT03288207) returned the GPS units to research staff members, GPS tracking was generally accepted. Pilot device testing revealed several concerns that will be addressed in this study. For instance, participants mentioned that they often forgot to charge the GPS device each night, as required by their limited battery life. They also sometimes forgot to bring the GPS with them when they left the house each morning.

For the overall study, we plan to address these device issues using the EMA app. The morning EMA prompt will include a message reminding participants to bring all of their devices, and the evening prompt will include a message reminding participants to charge their GPS units. These messages will hopefully curtail user errors and increase device compliance.

### Strengths, Limitations, and Expectations

#### Strengths

One strength of this study is that it focuses on both healthy White and African American adult women who reside in high- and low-SES neighborhoods in Washington, DC This study accounts for biological phenotyping based on age, race, and BMI to elucidate the differences in biomarkers of amygdala activity in relation to differential exposure to neighborhood social environment and PA. Furthermore, this pilot study may be one of the first to use three distinct geospatial wearable and activity monitoring devices for real-time assessment of environmental exposures via GPS, activity levels through accelerometers, and perceptions by the EMA smartphone app, followed by linkage to biomarkers of stress (ie, amygdala activity).

#### Limitations

This study had several limitations that need to be addressed. We may have some difficulties in recruiting African Americans residing in Ward 3, as the number of African American women living in Ward 3 is lower than that in Ward 5 in Washington, DC. However, our research group has strong relationships with organizations throughout Washington, DC, which will allow us to help with recruitment efforts in Ward 3. Furthermore, we will closely work with the recruitment liaison at the NHLBI at the NIH to resolve this issue. We should be able to recruit both White and African American female participants from Ward 5 from low-SES (household income of US $50,000-74,999 [about 15% of total residents in Ward 5]) to high-SES neighborhoods (household income of US $100,000-149,999 [about 15% of total residents in Ward 5]). In addition, this study has a cross-sectional design, thus limiting causal inference. Another limitation is that this study has a relatively small sample size (approximately 60 women) to assess differences in vascular function and immune cell activation based on exposure to adverse neighborhood social environment in Washington, DC. Note that the intent of this study is to establish feasibility and sample size needs for a larger study. We will also compare the extremes of neighborhood social factors to see if there are differences in vascular measures (eg, 5% highest poverty vs 5% lowest poverty) and assess racial differences in vascular inflammation and function within each ward. Finally, our analyses are at the person level, which do not intend to elucidate the within-day variability of PA and psychosocial factors in relation to biomarkers of stress. For a larger study, we plan to investigate the associations between within-day variability and outcomes.

#### Expectations

We anticipate that aim 1 will improve our current knowledge of the links between adverse neighborhood social environment features (eg, physical disorder) and stress-related neural activity (ie, amygdala activity), based on our prior experience demonstrating differences in amygdala activity between the DC-CHNA cohort and age- or sex-matched healthy volunteers [53]. Throughout this pilot study, we will disseminate the findings to the DC-CHOC members to gather input on the relevance of study findings to city practitioners and policy makers. These data will provide new insights into the role of the neighborhood social environment as a source of chronic psychosocial stress that can impact CV health.

In aim 2, we will be able to compare differences in vascular function between participants from Ward 3 (high-SES neighborhood) and Ward 5 (low-SES neighborhood). In addition, we will test the moderation effects of objectively measured PA using accelerometers because it is well known that PA influences vascular structure and function [76]. We expect that those living in adverse neighborhood social environments may have worse vascular function, which can be moderated by levels of PA. Those engaging in higher PA may have better vascular function, even if they reside in adverse neighborhood social environments.

In aim 3, we will be able to compare differences in immune activation between populations from two distinct neighborhood conditions (Wards 3 vs 5). We will also determine precise neighborhood social environment exposures within one’s residential and GPS activity spaces that relate to immune system activation, while adjusting for objective PA levels. On the basis of our previous data showing differences in inflammatory biomarkers between the DC-CHNA cohort and healthy volunteers [20], we expect that aim 3 will also be hypothesis-generating and will identify potential pathways involved in immune cell activation and function along the neural-hematopoietic-inflammatory axis, influenced by adverse environmental conditions.

### Conclusions

This pilot study will contribute to limited research on biomarkers of stress in relation to neighborhood social environment by applying geospatial methods and wearable devices, such as GPS, EMA, and accelerometers, to elucidate the mechanism by which adverse neighborhood social environment conditions impact the differences in stress-related neural activity by high and low SES in Washington, DC. If this pilot study can lead to an explicit understanding of biomarkers of stress, we may expand this study to other at-risk large populations in other areas of the Washington, DC, metropolitan area.

## Abbreviations

AIC: Akaike information criterion
CVD: cardiovascular disease
DC-CHNA: DC CV Health and Needs Assessment
DC-CHOC: DC Cardiovascular Health and Obesity Collaborative
EMA: ecological momentary assessment
FDG: fluorodeoxyglucose
GIS: geographic information system
IL: interleukin
IRB: Institutional Review Board
NHLBI: National Heart, Lung, and Blood Institute
NIH: National Institutes of Health
PA: physical activity
PET/CT: positron emission tomography-computed tomography
SES: socioeconomic status
